# A Review of Microfluidic Experimental Designs for Nanoparticle Synthesis

**DOI:** 10.3390/ijms23158293

**Published:** 2022-07-27

**Authors:** Adelina-Gabriela Niculescu, Dan Eduard Mihaiescu, Alexandru Mihai Grumezescu

**Affiliations:** 1 Department of Science and Engineering of Oxide Materials and Nanomaterials, Politehnica University of Bucharest, 011061 Bucharest, Romania; adelina.niculescu@upb.ro; 2 Department of Organic Chemistry, Politehnica University of Bucharest, 011061 Bucharest, Romania; danedmih@gmail.com; 3 Research Institute of the University of Bucharest—ICUB, University of Bucharest, 050657 Bucharest, Romania; 4 Academy of Romanian Scientists, Ilfov No. 3, 050044 Bucharest, Romania

**Keywords:** microfluidic devices, microfluidic channels, device geometry, microfluidic syntheses, microreactors, micromixers

## Abstract

Microfluidics is defined as emerging science and technology based on precisely manipulating fluids through miniaturized devices with micro-scale channels and chambers. Such microfluidic systems can be used for numerous applications, including reactions, separations, or detection of various compounds. Therefore, due to their potential as microreactors, a particular research focus was noted in exploring various microchannel configurations for on-chip chemical syntheses of materials with tailored properties. Given the significant number of studies in the field, this paper aims to review the recently developed microfluidic devices based on their geometry particularities, starting from a brief presentation of nanoparticle synthesis and mixing within microchannels, further moving to a more detailed discussion of different chip configurations with potential use in nanomaterial fabrication.

## 1. Introduction

Microfluidic technology has attracted increasing scientific interest in the last decades, gaining attention for numerous applications, especially in the fields of chemistry and biology [1,2]. Based on the use of miniaturized devices with channels and chambers of tens to hundreds of micrometers, microfluidic technology enables highly precise fluid manipulation. This feature is reflected in the ability of microfluidic devices to outperform conventional large-scale synthesis systems in terms of multistep control of the synthesis parameters, process reproducibility, ease of integration, and high throughput. By improving the synthesis conditions, microreactors are also appealing for performing complex reactions with superior control of kinetics and product characteristics. Thus, microfluidics has become a rapid, low-cost, reliable method for fabricating nanomaterials with fine-tuned properties and functions [1,3,4,5,6,7].

Syntheses carried out in microfluidic devices often employ multiple phases that are immiscible with each other (e.g., aqueous–organic liquids, gas–liquid reactions). Nonetheless, microfluidic mixing is not governed by the same laws as macroscale mixing, having a laminar regime in these small channels. To improve mixing efficiency in microreactors, altering their geometrical patterns is a simple and convenient solution. Therefore, numerous research studies focused on developing and evaluating a broad range of microfluidic designs for synthesizing materials of high quality [6,7,8,9].

In this respect, this paper begins by briefly discussing microfluidic nanoparticle synthesis and mixing. Further, there are reviewed, according to their geometry, the recently manufactured microfluidic devices with potential use in nanomaterial fabrication to present the state-of-the-art in the field and emphasize the versatility of these microreactors.

## 2. Nanoparticle Synthesis

Despite being still in its infancy, growing evidence shows the great potential of microfluidics in a plethora of applications, counting personalized medicine, disease diagnosis, chemical screening, cell culture, cell separation, cell treatment, drug screening, drug delivery, and DNA sequencing [10,11,12,13]. Moreover, the particles that can be obtained in microreactors can be further used in diverse areas, including electronics, energy, textiles, biotechnology, bio-imaging, bio-sensing, and gene delivery [14].

The small dimensions and unique geometries of microfluidic devices permit the use of smaller reagent volumes, more precise control of fluid mixing, efficient mass transport, improved heat transfer, and reduced reaction time, also allowing the possibility of process automation [15,16,17,18,19,20,21]. Thus, microfluidics holds the potential to overcome important drawbacks of scale-up reactors, such as the need for large spaces, expensive equipment, high-power consumption, alternation of synthesis conditions, insufficient control over mixing, complex stepwise operations, and safety concerns [18,22,23].

Consequently, microfluidic technology is increasingly used in synthesizing nanoparticles and carrying out various chemical syntheses. Compared to bulk methods, microfluidic syntheses permit the production of highly stable, uniform, monodispersed particles with tunable size and well-controlled features by simply controlling the geometry of the platform and the flow rates of involved fluids [1,14,15,18,19,22,23,24,25].

Regarding synthesis methods, two main types of microreactors can be distinguished according to their flow type. These are single-phase (continuous-flow microfluidics) and multi-phase flow (segmented flow or droplet-based flow microfluidics) devices, having different mixing patterns within the microfluidic channels. Briefly, the continuous flow regime implies mutual diffusion of reagents on the interphases, while segmented and droplet-based flows involve flow division into sections of limited volume within the device [23,26].

### 2.1. Continuous Flow

Microfluidic nanoparticle production most frequently revolves around continuous flow devices as this flow pattern offers simplicity, homogeneity, and versatility in controlling process parameters [23,27,28,29]. Such microfluidic systems suppose a laminar stream of two or more reagents and exploit the inherent features of the microchannel itself to combine the solvent and nanomaterials [5,26]. Thus, continuous flow is characterized by a lack of turbulence and a small Reynolds number (i.e., under standard conditions does not exceed 0.1) which translates to a very low mixing efficiency as fluid mixing mainly depends on diffusion. Nonetheless, these limitations can be overcome by inducing turbulence through bending/folding and stretching the microchannels [5,23,26,30,31].

Specifically, the geometry of single-phase microfluidic platforms can employ spiral or zigzag channels, embedded barriers, and other shapes or combinations between these variants. These passive mixing approaches are convenient to use as they do not require integrating additional complex parts within the device. However, for certain reactions (e.g., involving the mixing of fluids with high viscosity), sophisticated topology is insufficient and active components must be introduced into the system to enhance mixing efficiency [26].

### 2.2. Droplet-Based and Segmented Flow

Within droplet-based or segmented flow microfluidics, the use of immiscible phases leads to the appearance of discrete volumes in which the reagents and compounds are mixed in tiny, compartmentalized amounts. Specifically, the dispersed phase is restrained in the continuous phase, resulting in drops or segments that can be viewed as isolated microreactors. The generated drops of controlled size and shape do not interact with the channels of the device; in contrast, the formed segments are restricted by the channel walls. Nonetheless, both such systems offer a very fast and efficient homogenization process [5,26,32]. In more detail, multi-phase flow facilitates passive mixing as it enhances mass transfer, narrows the deviation of residence time, and limits reagents/products deposition on channel walls [23]. These aspects are further reflected in the generation of high-quality materials (reproducible and scalable particles with custom sizes, shapes, and morphologies). Nonetheless, several challenges exist also when using multi-phase flow systems, the most common drawbacks being the poor stability of droplets and the fact that droplets are never isolated (i.e., an extent of material exchange often takes place between droplets). These issues might or might not affect the desired outcome depending on what the device is used for [23,33]. However, the stabilization of droplet interfaces can be improved by adding surfactants [34].

In terms of geometry, droplets or segments can be formed by passive methods within microdevices based on three main microfluidic configurations, namely coaxial flows (co-flowing systems), flow-focusing reactors, and intersecting flows in a T-shape device (T-injector) [5,26]. In addition to device geometry, the channels’ dimensions and fluids’ flow rates need to be regulated to ensure precise monitoring and control over nanoparticle production through droplet microfluidics [33,35].

## 3. Mixing

The ability to thoroughly mix fluids is essential in microfluidic nanoparticle production. Thus, the literature focus has been noticed on various microfluidic mixing techniques. Micromixers may exist in two or three dimensions and are generally classified as active or passive [36,37].

Active mixing implies the use of an external force or an external physical field (e.g., magnetic, electrokinetic, acoustic, ultrasonic) to induce or accelerate the mixing phenomenon. Active mixers are usually more efficient than passive mixers, yet the need to integrate an external force or field in the platform makes it a less viable option for rapid prototyping and commercial translation, given the unwanted complexity and supplementary costs [36,37,38,39].

On the other hand, passive mixing is realized only by diffusion, being dependent on the area of contact between the fluids and the amount of time they are in contact [36]. Passive control methods often involve adjusting the contact angle with the channel surface and manipulating channel geometry to modify flow behavior, as the capillary force is difficult to maneuver once flow begins [40]. Thus, to disrupt the laminar flow, passive mixers require the use of special channel designs, relying on their geometry to stretch, fold, break, and split fluid flow to combine reagents by maximizing the contact area between the fluids and reducing the diffusion length [37,39,41]. Despite being simpler and more economical, passive mixers also present some drawbacks. Increasing the contact area of the fluids by lengthening the channel results in additional fluidic resistance to the channel, while increasing contact time by slowing the flow rate leads to a decreased throughput of the microfluidic system [36].

Passive micromixers used for fabricating nanostructures involve various geometries, including T-shaped mixing channels, coflowing junctions, hydrodynamic flow focusing, staggered herringbone mixer, two-layer crossing channel mixers, and 3D serpentine designs [39,42]. As planar passive micromixers were observed to not provide appropriate mixing efficiency over short channel lengths and wide ranges of Reynolds number, 3D micromixers started being regarded as a convenient alternative. Through fluid manipulation in a third dimension, there is an increase in fluid contact times, surface disruption, and a decrease in required channel length, leading to highly improved mixing efficiency. Nonetheless, the fabrication for 3D micromixers is more difficult than for planar microdevices, impeding their direct industrial translation [37].

## 4. Geometries

### 4.1. T-Type Microreactors

Planar T-shaped micro-junction represents the simplest and most used microfluidic geometry [1,43]. A T-type microreactor presents two channels positioned in a perpendicular manner: the main channel and an inlet channel [26]. In the standard configuration, the orthogonal channel contains the dispersed phase that intersects the main channel filled with the continuous phase, leading to droplets formation at the channels’ junction. By the addition of carefully chosen surfactants, T-type microreactors can generate oil-in-water and water-in-oil emulsions [1].

**Figure 1:**
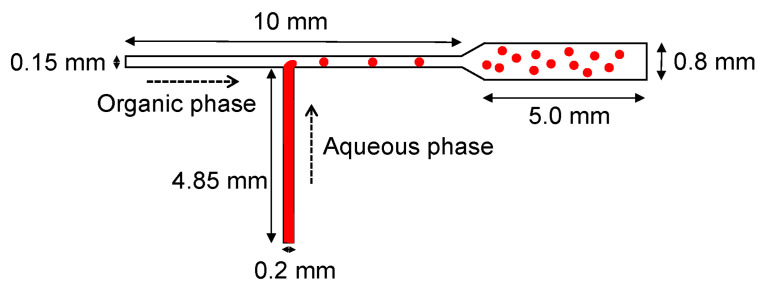
Schematic illustration of microdroplet formation in a T-shaped microfluidic channel. Reprinted from an open-access source [44].

For instance, Sasaki and Sugenami [44] have utilized a T-shaped microfluidic channel to form monodisperse microdroplets. The device was fabricated with the aid of a consumer-grade laser cutter with the design indicated in Figure 1. In the proposed device, the aqueous phase is pumped from the bottom of the T-shape microchannel, while the organic phase is pumped from the left side, leading to the generation of water-in-oil type microdroplets. The authors concluded that this method could be employed for creating microdroplets with high concentrations of macromolecules as in cells.

**Figure 2:**
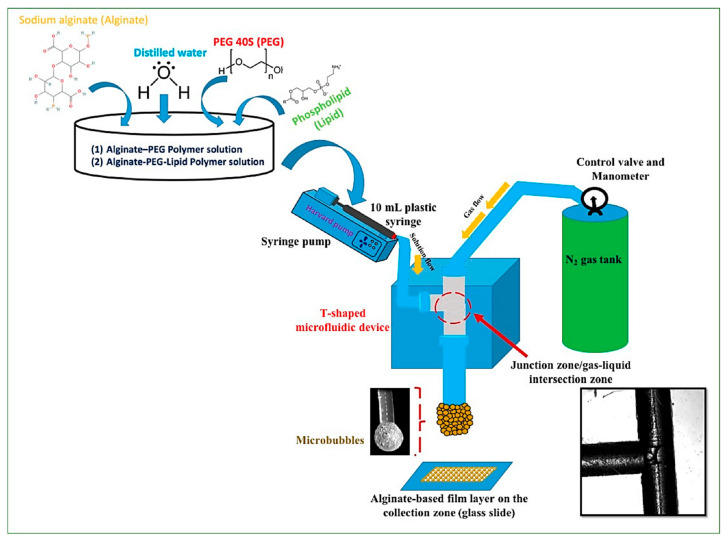
Schematic representation showing the T-shaped microfluidic junction processing of an alginate-based porous film formation from bubble bursting. Reprinted from an open-access source [45].

Alternatively, Mutlu et al. [45] used a T-shaped microfluidic junction device (Figure 2) to create highly monodisperse porous alginate films from the bubble bursting. Their microfluidic platform was made of polymethyl methacrylate through CNC machining. It consisted of the inlet (15 cm) and outlet (5 cm) Teflon capillaries with an inner diameter of 200 μm. Additionally, the experimental setup included a gas cylinder to provide nitrogen to the vertical capillary for producing monodisperse bubbles and a disposable plastic syringe connected to a syringe pump to ensure polymeric solution flow to the horizontal capillary.

**Figure 3:**
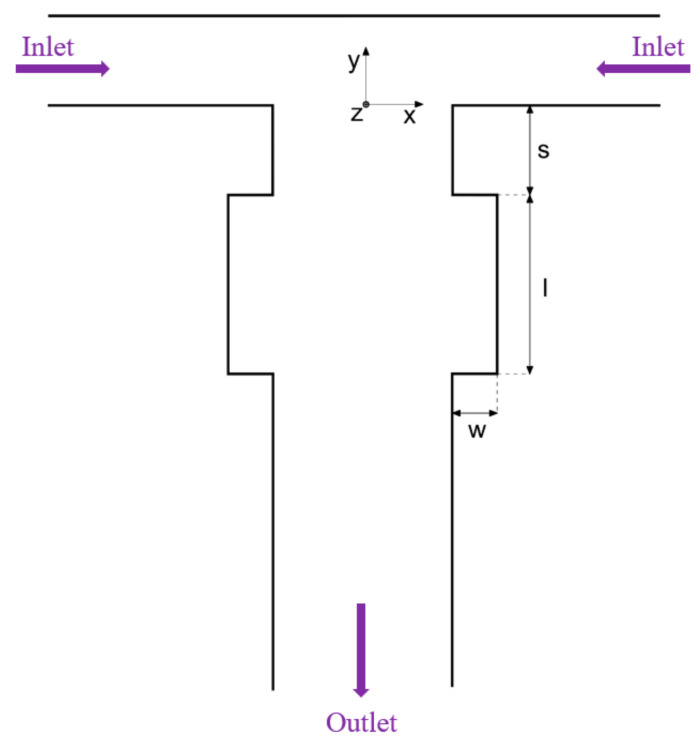
Sketch of the geometry proposed by Antognoli et al. Adapted from an open-access source [43].

More recently, Antognoli and colleagues [43] investigated whether the introduction of one pair of small rectangular cavities in the lateral walls downstream of the T junction would enhance the mixing efficiency of the device. The authors proposed a device (Figure 3) with inlet channels of the T-shaped confluence region of 1 mm width and 40 mm length and an outlet channel of 2 mm width and 60 mm length. The height of the channels is 1 mm, and the hydraulic diameter is 1.33 mm. The researchers concluded that the modification of the outlet channel improves mixing efficiency while preserving the simplicity of T-shape geometry without significant pressure drops. Moreover, the width of the mixing channel (compared to the width of the inlet channels) is an essential factor for the onset of different flow regimes as it increases the Reynolds number.

### 4.2. Y-Type Microreactors

**Figure 4:**
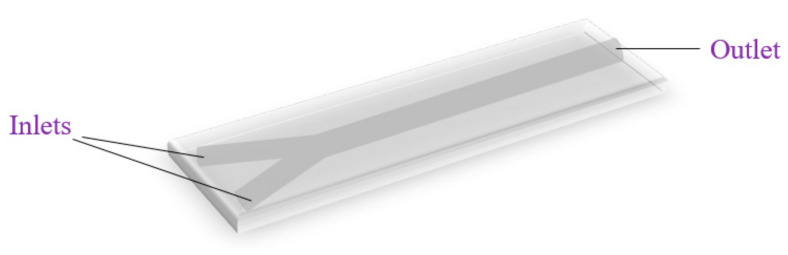
Schematic representation of a typical Y-type microreactor.

A Y-type device represents another simple microfluidic geometry for generating nanostructures. Such a platform supposes the existence of two inlets and one outlet (Figure 4). The two reagent-containing fluids are introduced through each inlet, further flowing through the two channels up to the intersection, where they combine and mix throughout the length of the straight main channel until reaching the outlet [6,40,46].

As an example, Ballacchino et al. [6] have used a Y-type microreactor for fabricating liposomal nanoparticles. The fluids they utilized were a stream of lipid in an alcohol solution and a stream of aqueous solution (i.e., phosphate-buffered saline, PBS). Liposome synthesis is achieved by the intersection of the two channels and the diffusion process occurring at the fluids interface. Thus, in the straight main channel, precipitation of the lipids in an aqueous solution is observed to form micelles first and liposomes after.

Given the simplicity of this geometry, it is often employed in association with other channel shapes, as it is further described in Section 4.8. Combined geometries.

### 4.3. Flow-Focusing Microreactors

Planar flow-focusing microfluidic systems represent another commonly approached type of geometry for chemical syntheses. In this configuration, the dispersed phase flows through the middle channel while the continuous phase is introduced through the two outside channels. Then, both phases are forced through the orifice located downstream of the three channels [1]. Thus, drops or segments are produced by the dispersed phase’s hydrodynamic focusing (shearing) by the continuous phase [26]. Moreover, droplet size, velocity, and frequency can be adjusted by controlling flow rates, inlet pressures, phase viscosities, and orifice size [1]. The versatility of these devices and the great control over reactant mixing render these microfluidic platforms suitable for synthesizing various nanostructures, including polymer beads [32,47,48], metal oxide nanoparticles [49,50], and spin crossover nanomaterials [51].

**Figure 5:**
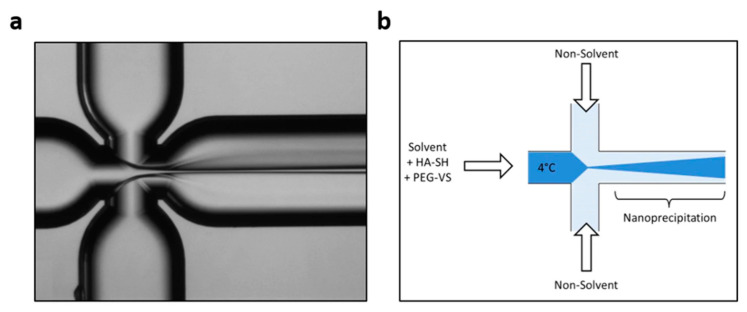
Schematic illustration of the microfluidic experimental setup used by Tammaro et al. (**a**) Optical Fluorescence Microscopy Image of Flow-Focusing pattern; (**b**) Qualitative Illustration of crosslinking strategies. Reprinted from an open-access source [52].

For instance, Tammaro et al. [52] have used an X-junction flow-focusing microreactor to crosslink hyaluronic acid (HA) and polyethylene glycol (PEG) (Figure 5). For this purpose, the researchers injected an aqueous solution of thiolated HA and PEG-vinyl sulfone in the middle channel and a non-solvent (i.e., pure acetone) in the side channels. For this synthesis, a quartz microfluidic platform called “Droplet—Junction Chip” was used, having channels of 190 μm depth and 390 μm width and an internal surface coated by a hydrophobic material. Even though this device has two separate droplet junctions, for the nanoprecipitation reaction, only one was employed.

Lee et al. [32] employed a flow-focusing microfluidic configuration with homogeneous channel height (i.e., 50 μm) to fabricate alginate droplets with front–back asymmetry. The authors have studied appropriate conditions for anisotropic gelation and concluded that it could be induced by droplet fusion during a dripping regime around the channel junction.

**Figure 6:**
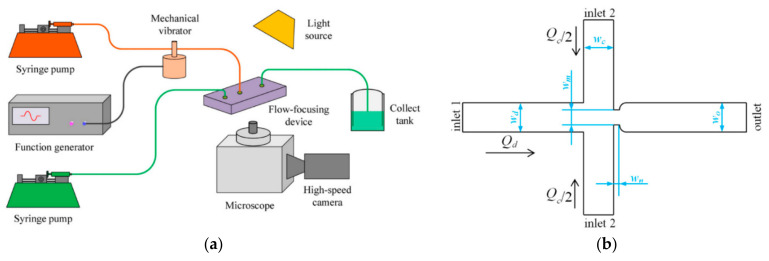
(**a**) Schematic diagram of experimental setup for droplet generation in flow-focusing microfluidic chip with external mechanical vibration. (**b**) Schematic diagram of the flow-focusing microfluidic chip. Reprinted from an open-access source [53].

Differently, Yin et al. [53] have studied droplet generation in a flow-focusing microfluidic device coupled with an external mechanical vibration system (Figure 6a). Nonetheless, the geometry of the channels is similar to the above-described models, as indicated in Figure 6b, where inlet one is the entrance point of the dispersed phase, inlet two is the entrance point of the continuous phase, and the outlet represents the exit towards the collecting tank. All channels have a width of 100 μm and a height of 38 μm, while the connecting aperture has a width of 50 μm and a length of 25 μm.

**Figure 7:**
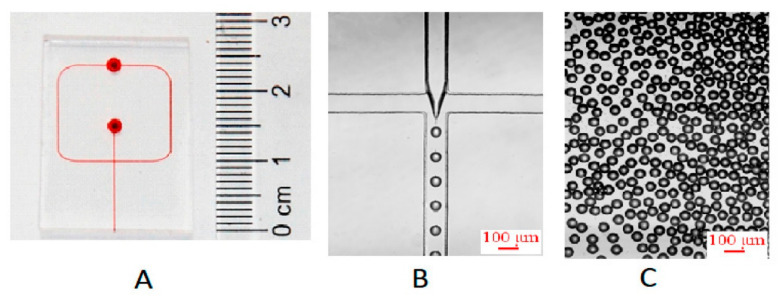
(**A**) Photograph of the polydimethylsiloxane (PDMS) microchip. Channel and reservoirs were full of red ink for visualization. (**B**) Micro photo of the process of microdroplets preparation at cross-junction channel. The velocity of the aqueous phase and oil phase was 14 and 80 μL/min, respectively. (**C**) Microphoto of microdroplets off-chip. Reprinted from an open-access source [54].

Alternatively, Hong and colleagues [54] have used a flow-focusing microchip to generate uniform microdroplets containing silver seeds and a growth solution (Figure 7). The channels had equal width and height (i.e., 100 μm). The dispersed phase (silver nitrate, trisodium citrate, silver seeds, and pure water) and the continuous phase (liquid paraffin) were delivered by two syringe pumps into the micro-platform through polytetrafluoroethylene tubes with an inner diameter of 0.5 mm.

**Figure 8:**
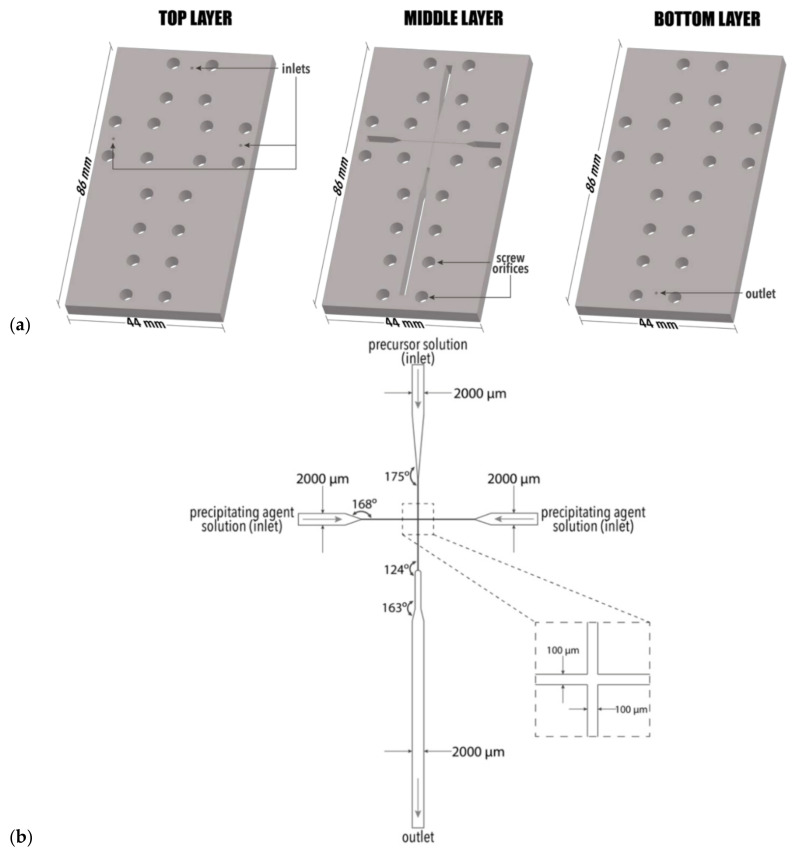
(**a**) Configuration of the three layers comprising the microfluidic device and (**b**) Configuration of the cross-junction channel within the middle layer of the device utilized by Chircov et al. Reprinted from an open-access source [50].

Another interesting microfluidic device configuration is proposed by Chircov et al. [50]. The research group utilized a 3-layered chip configuration, as illustrated in Figure 8, for synthesizing magnetite nanoparticles. The involved geometry allowed for a highly controlled co-precipitation process, precise tailoring of product characteristics, and, subsequentially, synthesis of highly uniform nanoparticles.

On a different note, Gao and Chen [55] have reported the use of a flow-focusing microfluidic glass capillary assembly for encapsulating solid cores to prepare double emulsions. The chosen geometry allowed the controlled and reliable formation of double emulsion droplets under a wide operating range of flow rates.

### 4.4. Co-Flowing Microreactors

As opposed to flow-focusing devices, in co-flowing microreactors, the dispersed and continuous phases are delivered from the same direction. In more detail, the dispersed phase is injected into a small capillary centered inside a larger-diameter channel filled with the continuous phase flowing in parallel. Thus, the continuous phase surrounds the dispersed phase, making the viscous shear force stronger with the increase in dispersed phase diameter and leading to its fracturing into monodispersed droplets or a liquid jet. The regimens differ depending on flow rates, as follows: at low flow rates, dripping is predominant, producing spherical droplets; at high flow rates, jetting is predominant, leading to droplets formation via the breaking of a thin stream of dispersed phase farther downstream due to convective instabilities [1,26,46].

Particles of various shapes and sizes can be obtained with the aid of co-flowing microfluidic devices. For instance, Badali et al. [56] have recently fabricated cell-laden silk–fibroin–phenol microparticles through such a device. In this respect, the researchers delivered the aqueous silk–fibroin–phenol solution through the inner channel of the co-flow system and liquid paraffin saturated with hydrogen peroxide through the outer channel.

A different example is offered by Cai and colleagues [57], who have used a co-flow microreactor for synthesizing bullet-shaped microparticles. For this purpose, the researchers employed two 3-cm-length-cylinder capillaries with an inner diameter of 550 μm and an outer diameter of 960 μm as injection tube and transition tube, and another 16.8 cm-length-cylinder microcapillaries with an inner diameter of 300 μm and outer diameter of 960 μm as the collection tube. This geometry allowed for the fabrication of monodispersed particles with controlled structures, obtained from droplets deformed by fluid shear stress and spatial confinement of collection tubes as templets.

**Figure 9:**
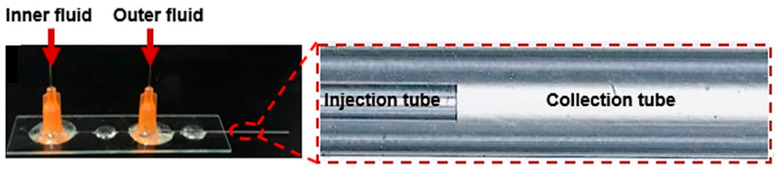
Microfluidic device with a co-flow structure developed by Xia et al. Reprinted from an open-access source [58].

Another co-flow microfluidic device is described by Xia et al. [58]. The authors used two cylindrical glass capillaries as injection and collection tubes. The injection tube had an inner diameter of 300 μm and was coaxially positioned inside the collection tube that had a 900 μm inner diameter. Two glass tubes were further fixed on the glass sheet with the aid of epoxy glue, and their entrance was connected with a dispensing needle, as displayed in Figure 9.

**Figure 10:**
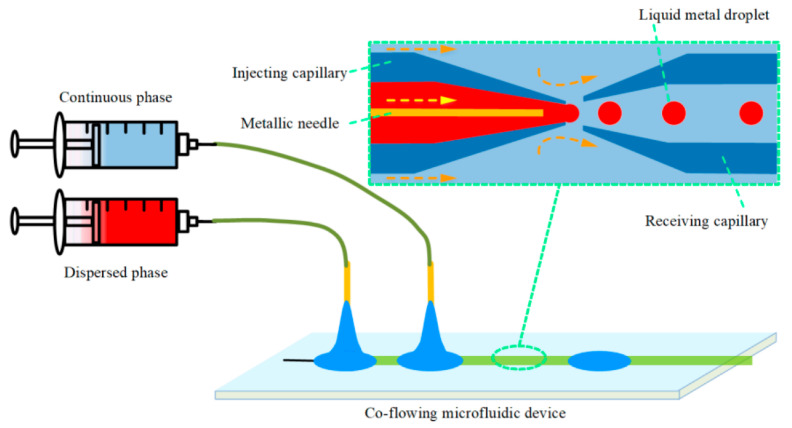
Schematic representation of the micro-needle induced co-flowing microfluidic experimental setup. Reprinted from an open-access source [59].

Hu et al. [59] have performed a numerical investigation on a co-flowing capillary device with a micro-needle in the inlet capillary (Figure 10). The microfluidic device was fabricated by assembling two tapered glass capillaries inside a square channel, concentrically aligned, with an axial spacing of 100 μm. The metallic needle was placed into the inlet capillary along the orifice axis, with a 175 μm transverse distance between the micro-needle tip and the center of the capillary orifice.

**Figure 11:**
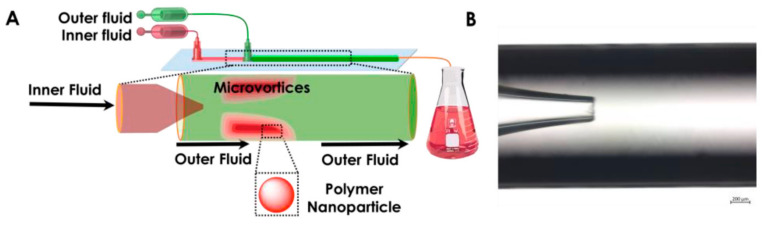
(**A**) Schematic representation of 3D co-flow microfluidics and (**B**) Digital view of the inner and outer capillary. Reprinted from an open-access source [60].

A different system is proposed by Ma and colleagues [60], who have employed a 3D co-flow microfluidic device for synthesizing polymer particles. For the nanoprecipitation reaction, the authors used two miscible liquids: an aqueous solution of a surfactant (outer continuous fluid) and an ethanol solution of the polymer (inner dispersed phase). The inner and outer fluids were independently pumped into the device, and the organic phase exited as a jet from the inner capillary, quickly mixing with the outer fluid, as depicted in Figure 11.

### 4.5. S-Shaped Microchannels

Mixing can also be enhanced by integrating S-shaped channels within the reaction zone of the microfluidic platform. For example, ye and colleagues [61] have reported using an S-shaped micromixer to synthesize gold nanobipyramids with controllable morphology. The microfluidic chip was made of glass wafers and a polydimethylsiloxane (PDMS) layer with well-defined holes for tubing connection. The layers were bonded by oxygen plasma to create the overall device having S-shaped microchannels of 100 μm depth and 200 μm width.

Alternatively, Zhou et al. [4] proposed the use of several parallel S-shaped channels for mixing fluid to improve the quality of nano-catalysts synthesis. The authors created a metal plate chip with symmetric dendritic bifurcation, increasing the number of parallel channels for the same set of inlets and a collection chamber for the outlet zone to reduce the resistance of the reaction channels. In order to ensure proper mixing, each precursor fluid is separately diverted into a parallel series of flows before combining with the other fluid; the use of multilayered channels achieves this. According to the authors, this design enhances mixing by generating chaotic convection, further leading to increased uniformity of the products. Moreover, this strategy holds promise for mass production as it is simple, takes up a small volume, and is easy to integrate.

Clark et al. [62] suggested an interesting alternative, who performed experimental simulations and experimental studies on non-rectangular cross-sections in serpentine Dean flow micromixers. The researchers showed that this is a viable strategy for improving mixing performance at Reynolds numbers as low as 20, given that optimized designs present quick lamination after the first mixing unit due to chaotic advection onset.

**Figure 12:**
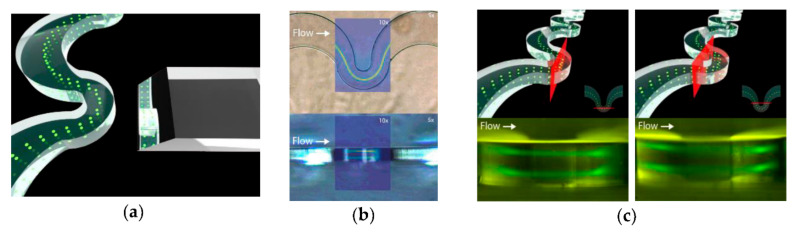
(**a**) Render that illustrates the position of the lateral mirror with respect to the serpentine; (**b**) Zenithal (top) and lateral (bottom) views of the serpentine with an overlapped fluorescence streak image (false color) from inertially focused particles at a flow rate of 130 μL/min; (**c**) The limited depth of field of the employed objective allows the streak of particles reflected in the mirror to focus on different focus planes (red planes). Reprinted from an open-access source [63].

On a different note, Pedrol and colleagues [63] have studied the dynamics of spherical particles in an asymmetric serpentine (Figure 12). The authors constructed a PDMS microfluidic device with the aid of conventional photolithography techniques. In addition to the conventional channels, the designed platform also incorporated cavities for the insertion of micromachined glass mirrors oriented at 45° near the serpentine so that their fields of view covered the entire height of the channel. In this manner, the scientists were able to visualize the two stable trajectories of inertially focused fluorescent polystyrene particles.

### 4.6. Staggered Herringbone Micromixer

Staggered herringbone microfluidic chips are an appealing configuration for ensuring a rapid mix of liquids. Through their specific V-shaped ridges on the bottom of the channels, these micromixers can alleviate the low Reynolds number problem by introducing transverse flow patterns that fully mix the reaction solution and enhance the diffusion of substances in the system [39,64].

For instance, Chiesa et al. [65] have utilized a staggered herringbone micromixer to synthesize tripolyphosphate crosslinked chitosan nanoparticles through an ionic gelation reaction. The geometry of the system consisted of a repeated pattern of grooves on the bottom of the microchannel, which can provide a rapid, chaotic mixing and thus induce the stretching and folding of the fluid under laminar flow.

**Figure 13:**
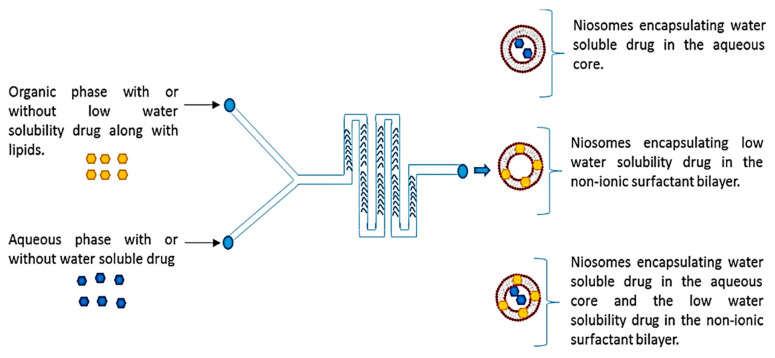
Schematic representing the microfluidic chip structure is having a staggered herringbone micromixer and the whole process of niosome formation. Reprinted from an open-access source [66].

Staggered herringbone micromixers can also be employed in synthesizing niosomes, as demonstrated by Joshi et al. [66]. The authors reported the use of NanoAssemblr^TM^ benchtop with 300 μm channels (Figure 13), which they introduced through one inlet garcinol dissolved in the organic phase and through the other inlet metformin dissolved in PBS.

### 4.7. Other Geometries

Scientists around the world have also developed various other geometries in an effort to create devices with enhanced mixing efficiency that would lead to the synthesis of nanomaterials with precisely tailored characteristics.

**Figure 14:**
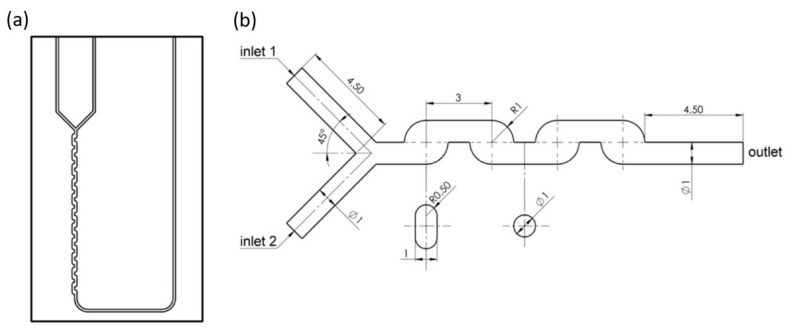
(**a**) Schematic representation of the microfluidic platform and (**b**) geometrical model of the chicane micromixer designed by Khaydarov et al. Reprinted from an open-access source [67].

One interesting geometry is proposed by Khaydarov and colleagues [67], who have developed a micromixer with a chicane mixing channel structure (Figure 14). This device imposed a convective mixing, with swirling and recirculation being two special cases of the process. Moreover, the asymmetrically formed channel provides a greater effect than a symmetrical one as the high quantity of curvatures through which the fluid passes increases the vorticity of the flow. Despite the advantage of improved mixing patterns, this type of channel brings higher costs than other established platforms, such as T-type microreactors or serpentine micromixers.

Alternatively, Tomeh et al. [68] have used a new type of microfluidic design: a swirl mixer. According to the authors, this novel design provides a high production rate, reproducibility, and precise control over particle size. The experimental chip was used to synthesize silk nanoparticles and lipid nanoparticles, allowing tuning of the mean size and size distribution through the variation of multiple processing parameters. The proposed micromixer could generate three types of flow (laminar, transitional, and turbulent flows) depending on the total flow rate applied, could achieve a higher flow rate (>300 mL/min) than many commercially available designs, and could be easily disassembled to allow for channel cleaning and sterilizing. Moreover, the same research group reported the use of swirl mixers for fabricating curcumin-loaded liposomes [42]. The enhanced mixing performance of the device at transitional and fully turbulent regimes ensured a short mixing time that further favored fast and simultaneous self-assembly of the components.

A different microfluidic system designed is proposed by Hao et al. [69]. The scientists used a five-run spiral-shaped microchannel with two inlets and one outlet for the controllable synthesis of mesoporous silica nanofibers. The chip was made of PDMS through soft lithography, and the channels had a 500 μm width and 50 μm height. The smallest spiral diameter was 5.25 mm, then increased from 11.0 mm to 22.2 mm with an increment of 1.4 mm for each half run. A year later, the same research group optimized the device for a different synthesis [70]. The authors reduced the number of runs in the spiral to two and used the new design for producing hollow spherical silica with hierarchical sponge-like pore sizes.

**Figure 15:**
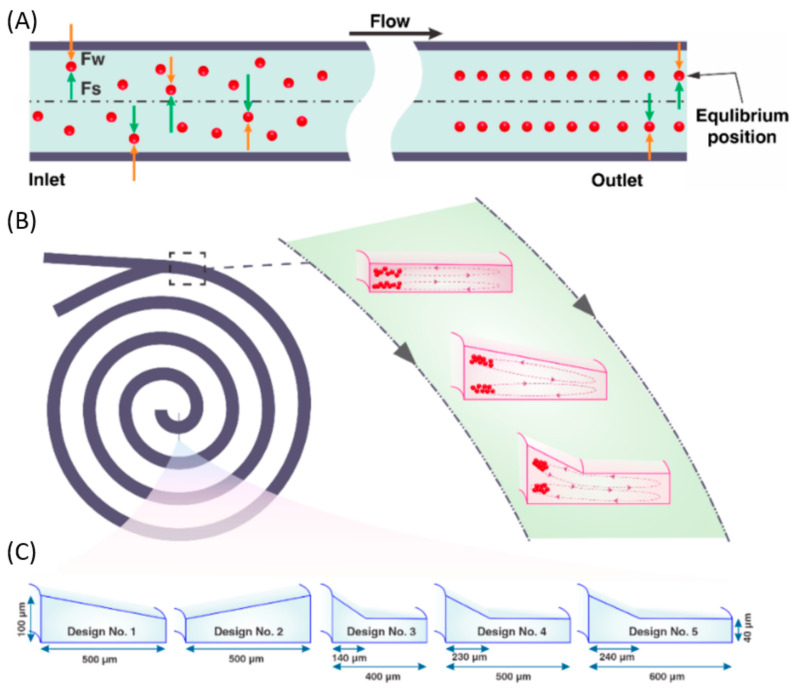
(**A**) The schematic illustration of particle focusing in straight rectangular microchannels. (**B**) Schematic of spiral microchannels. (**C**) Schematic of a cross-sectional view of the channels used in the work of Mihandoust et al. Reprinted from an open-access source [71].

Several other spiral designs have been developed by Mihandoust et al. [71], who have created passive microfluidic concentrators with complex cross-sectional shapes (Figure 15). All microchannels have spirals with 4.5 loops, while their cross-sections vary from inward sloping trapezoid to outward sloping trapezoid and the complex cross-section combines an outer part and an inner part. The outer part is rectangular, and the inner part is an inward sloping trapezoid. The authors concluded that incorporating trapezoidal and rectangular shapes into one shape confines the Dean vortices in the trapezoidal region, leading to an efficient focusing of particles and high throughput.

### 4.8. Combined Geometries

In addition to the above-discussed individual configurations, several geometries can be combined within the same chip as a way to enhance mixing performance, improve control over reaction parameters and obtain the desired nanostructures with higher throughput.

For instance, Kim and colleagues [72] have used a multiple repeated T-junction breakup microfluidic filter device in order to fabricate microspheres from premixed emulsions. This design helped droplet splitting, as microdroplets divided symmetrically at the T-junction in the microchannel. When the number of filters increased, droplet diameter decreased, and droplet size distribution significantly improved. Another observation is that microdroplet diameter significantly increases with the increase in emulsion viscosity.

Alternatively, Baydir and Aras [73] combined narrow channel tubular reactors of different diameters with t-type mixing cells of different diameters, studying their effects on biodiesel production. These configurations allowed for improved mixing and led to obtaining a high percentage of fatty acid methyl esters in a short residence time.

Wu et al. [74] have also included T-junctions in their device. Specifically, the authors created a droplet-based microreactor with two T-junctions and a tube for producing ficin-capped gold nanoclusters. The as-described microfluidic platform allowed for the rapid fabrication of nanomaterials with good polydispersity.

**Figure 16:**
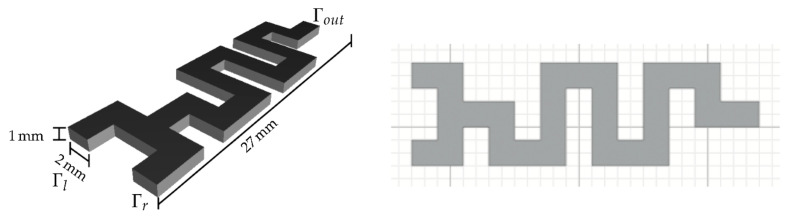
Geometry of the micromixer investigated by Jeßberger et al. (**Left**) Below in the picture are the two inlets Γl, Γr and the T-junction. On the top, we find the outlet Γout. (**Right**) Dimensions of the channel in the xy-plane on a grid with width 1 mm. Reprinted from an open access source [75].

Jeßberger et al. [75] have incorporated a T-junction into a different design. The scientists created a micromixer with two inlets, a T-type intersection, a meandering channel, and one outlet with the sizes and placement schematically represented in Figure 16. The channel has a cross-section of 1.0 × 10^−3^ m × 2.0 × 10^−3^ m and a total length of 2.7 × 10^−2^ m. The multiple 90° angle deflections significantly improved the mixing quality of the microfluidic system.

**Figure 17:**
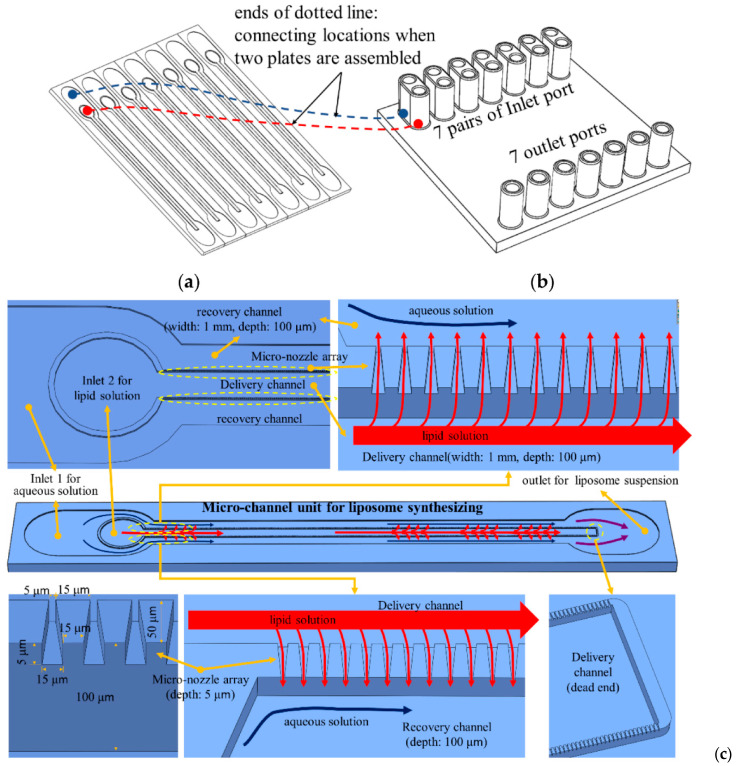
Microfluidic device designed by Woo et al.: (**a**) microchannel plate; (**b**) cover plate. (**c**) Schematic representation of the unit liposome-synthesizing microchannel. Reprinted from an open-access source [76].

A different approach was taken by Woo and colleagues [76], who used a device made of a micro-channel plate (Figure 17a) and a cover plate (Figure 17b) with inlet and outlet ports in order to synthesize liposomes. Moreover, the microchannel unit also included a micro-nozzle array, a delivery channel, and two delivery channels, as depicted in Figure 17c. Each micro-nozzle array connects outside with a recovery channel, thus leading to the formation of multiple T-junctions that help in the mixing process of the aqueous and lipid solutions within the recovery channel. Further downstream, two recovery channels merge into a single port to collect liposome suspension into a bottle through a tube.

**Figure 18:**
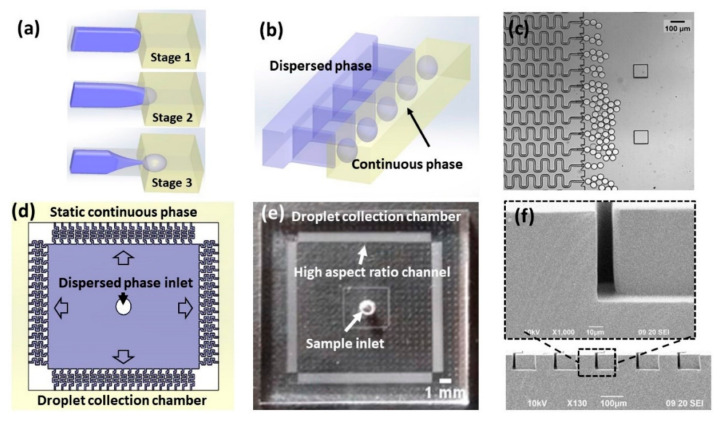
Schematic diagram and photographs of the high-aspect-ratio-induced self-breakup (HIDS) device: (**a**) Schematic representation of the droplet breakup process by Plateau–Rayleigh instability in a single HIDS structure where the dispersed phase (purple) is confined in an energy-unfavorable shape; (**b**) Schematic representation of the parallel integration of an array of the HIDS generators. (**c**) A micrograph of parallelized HIDS generators and generated monodispersed droplets. (**d**) A layout of a parallelized generator device indicating the sample applied at the center and emulsified into the continuous phase through the HIDS generators and collected in the droplet collection chamber in the peripheral. (**e**) A photograph of the device with a simple sample inlet and HIDS generators is indicated by arrows. (**f**) SEM image of the cross-section of the channels with a width of 13 μm and height of 65 μm. Reprinted from an open-access source [77].

Differently, Chung et al. [77] created a high-throughput droplet generator by parallelization of high aspect ratio rectangular structures (Figure 18). The multilayered device enabled the simple and scalable generation of uniform droplets without requiring strict control of the external flow conditions. The device comprises 1200 parallelized generators that can produce monodisperse droplets at a frequency of 25 kHz. The authors concluded that this approach is versatile for fabricating a wide range of functional materials for large-scale applications.

**Figure 19:**
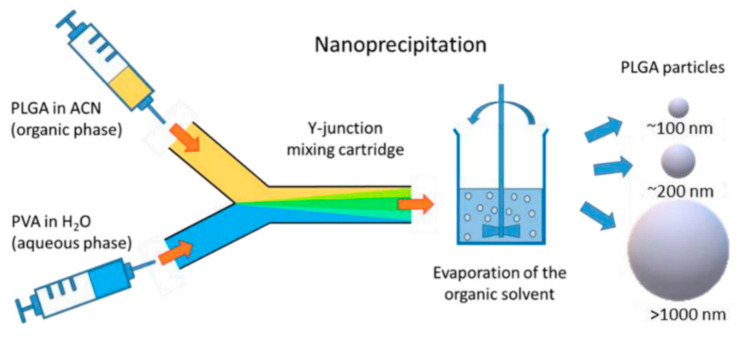
Schematic representation of the particle generation process via nanoprecipitation method using a Y-junction mixing cartridge. ACN: acetonitrile; PLGA: poly(lactic-co-glycolic acid); PVA: polyvinyl alcohol. Reprinted from an open-access source [78].

Another example is offered by Operti et al. [78], who have combined the benefits of a Y-type microreactor with those of staggered herringbone ridges for fabricating poly (lactic-co-glycolic acid) particles (Figure 19). This device allowed the highly controllable synthesis of particles with ~100 nm, ~200 nm, and >1000 nm diameter by simply modifying flow and formulation parameters.

**Figure 20:**
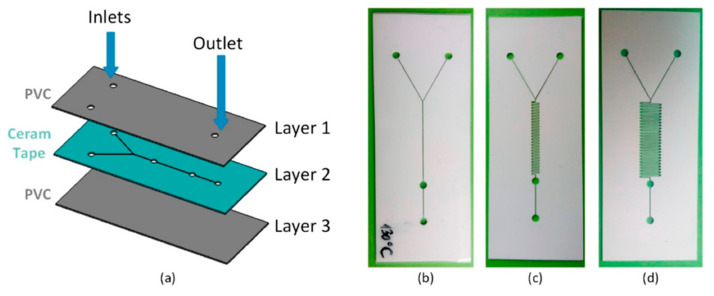
Microfluidic chips fabricated using the hybrid technology proposed by Kojic et al. (**a**) 3D model of the microfluidic chip (Layer1-PVC, Layer2-Ceram Tape, and Layer3-PVC), (**b**) simple channel, (**c**) short serpentine, and (**d**) long serpentine. Reprinted from an open-access source [79].

Kojic et al. [79] have also taken into consideration the advantages of Y-type microchannels and combined them with serpentine micromixers. Their proposed design consists of three layers, as illustrated in Figure 20. In all the developed microfluidic platforms, the channel width was 200 μm, whereas the holes for inlets and outlets had a diameter of 2 mm. The microfluidic devices were fabricated with the help of the xurography technique and a laser micromachining process. Thus, they can be obtained in a cost-effective manner while also resisting 3000 times higher flow rates than chips fabricated through the standard xurography technique.

**Figure 21:**
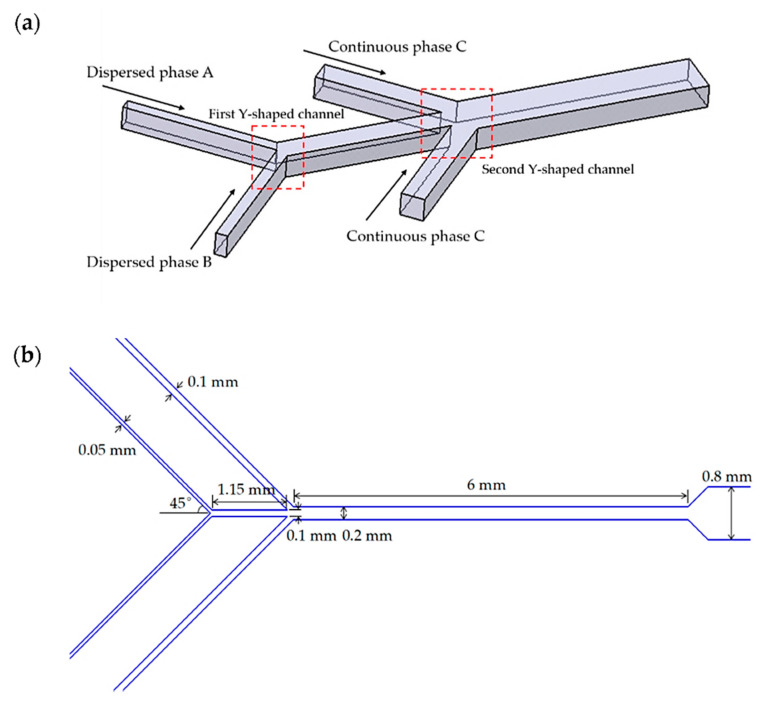
(**a**) Schematic illustration of the double Y-shaped channel used in the manufacture of Janus droplets; (**b**) Specific dimensions of each channel. Reprinted from an open-access source [80].

Another example of an improved Y-type microreactor is offered by Bai et al. [80], who created a double Y-shaped microfluidic channel able to generate Janus droplets. Their configuration comprises four inlets (two for the dispersed phase and two for the continuous phase) and one outlet, as represented in Figure 21. The inlet channels form angles of 45° with the main channel. For the dispersed phase, the channels have an inlet width of 50 μm and an intersection width of 100 μm, while for the continuous phase, these dimensions are double. The depth of the channel is equal throughout the entire microfluidic system (i.e., 100 μm).

One more example of an atypical microfluidic platform has been developed by Chen et al. [81]. The authors designed three-inlet planar mixing geometry, which they named “cascaded splitting and recombination” (C-SAR). The channel topography resembles cross-type microreactors, with the exception of the main channel exhibiting numerous asymmetric arranged triangular baffles in the mixing region. This design was noticed to create chaotic advection and enhance mixing, i.e., 90% mixing efficiency in a wide range of Reynolds numbers (34.6–150), being an attractive tool for studying biomacromolecular dynamics.

**Figure 22:**
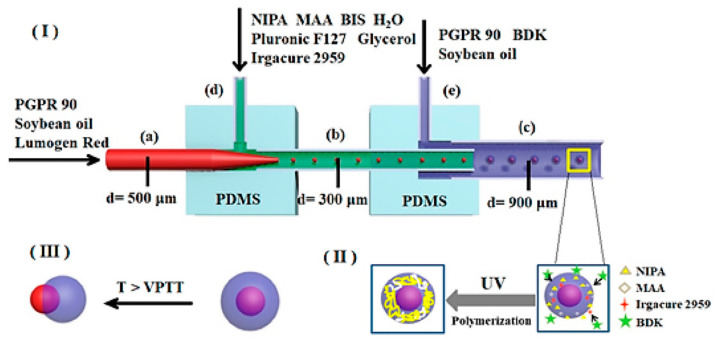
Schematic representation of microcapsules production via the microfluidic device used by Wang et al. (**a**) Glass capillary with an inner diameter of 500 μm; (**b**) Glass capillary with an inner diameter of 300 μm; (**c**) Collection tube; (**d**,**e**) Needles for fluid introduction. (**I**) Formation of O/W/O emulsion droplets on the chip; (**II**) UV-initiated polymerization of collected O/W/O emulsion droplets to form P(NIPA-*co*-MAA) microcapsules; (**III**) Thermo-triggered release behaviors of P(NIPA-*co*-MAA) microcapsules. Reprinted from an open-access source [82].

An interesting geometry has also been developed by Wang et al. [82]. The authors employed a double coaxial microfluidic device to synthesize thermos-triggered-releasing microcapsules for liposoluble drug delivery. Their device consisted of two PDMS connectors, three capillaries, and two needles, with the dimensions and placement illustrated in Figure 22.

**Figure 23:**
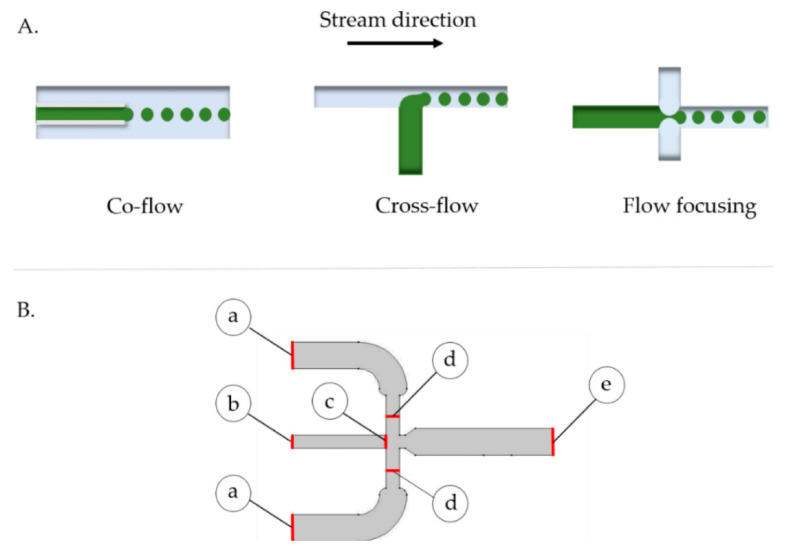
(**A**) Schematic representation of microfluidic system arrangements for microcapsule generation. The continuous phase is shown in blue, while the discrete phase is in green. (**B**) 2D geometry of the droplet generation junction in the designed microfluidic system chosen by Campaña et al. (a) Continuous phase inlet of 2 mm, (b) dispersed phase inlet of 1 mm, (c) initial interface of 1 mm, (d) continuous phase inlet of 1 mm and (e) flow outlet of 2 mm. Reprinted from an open-access source [83].

Alternatively, Campaña et al. [83] approached microfluidic synthesis for encapsulating the fungal lactase from *Pycnoporus sanguineus* CS43 in alginate microcapsules. For this purpose, the authors explored the design, manufacture, and characterization of a low-cost microfluidic system, the analyzed devices including geometries where the continuous and discrete phases come into contact in co-flow, cross-flow, and flow-focusing arrangements (Figure 23). Out of these possibilities, the flow-focusing microreactor type was chosen for fabricating polymeric capsules, given its enhanced control over microcapsules size by adjusting the last section’s channel width.

**Figure 24:**
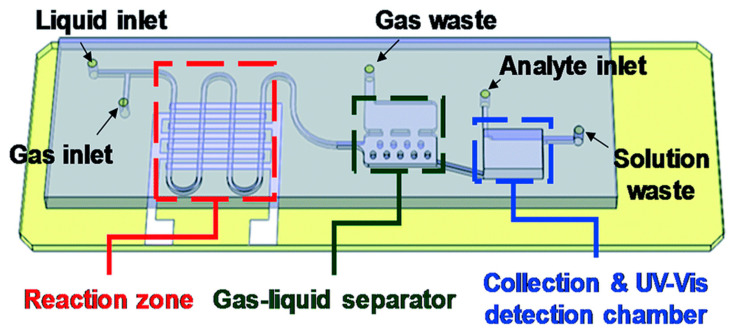
Schematic representation of the microfluidic platform designed by Li and Lin. Reprinted from an open-access source [84].

A distinct chip design was elaborated by Li and Lin [84], who integrated three different sections into the platform, each having a specific geometry (Figure 24). These sections are a serpentine micro plasma channel for droplet-based gold nanoparticle synthesis, a gas/liquid separation chamber, and a UV-Vis detection chamber, placed in this order. Thus, the device integrates on-demand gold nanoparticle synthesis using atmospheric pressure helium plasma and on-site detection of mercury ions in aqueous solutions.

**Figure 25:**
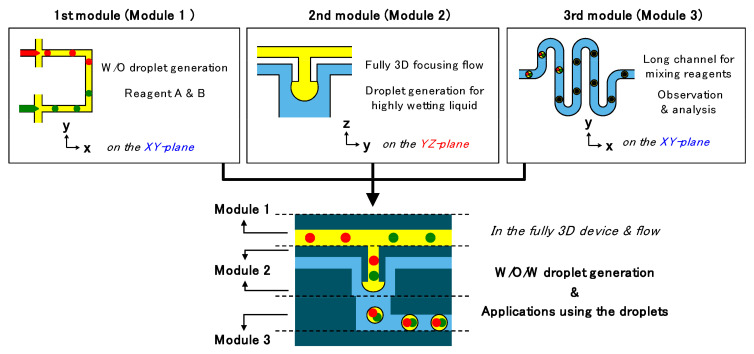
Schematic representation of the device formed by module integration; function and working plane of each module and fully integrated device developed by Yoon et al. Reprinted from an open-access source [85].

Yoon et al. [85] have proposed using three independent modules to create a complex 3D microfluidic platform (Figure 25). The final device combines the modules in such a manner that channels are placed both horizontally and vertically. Module 1 is intended for water-in-oil droplet formation; module 2—oil-in-water droplet formation; and module 3—mixing and observation. The designed system allowed the one-step generation of water-in-oil-in-water droplets without requiring partial treatment of the PDMS channel surface using separate modules for generating water-in-oil droplets on the horizontal plane and oil-in-water droplets on the vertical plane.

**Figure 26:**
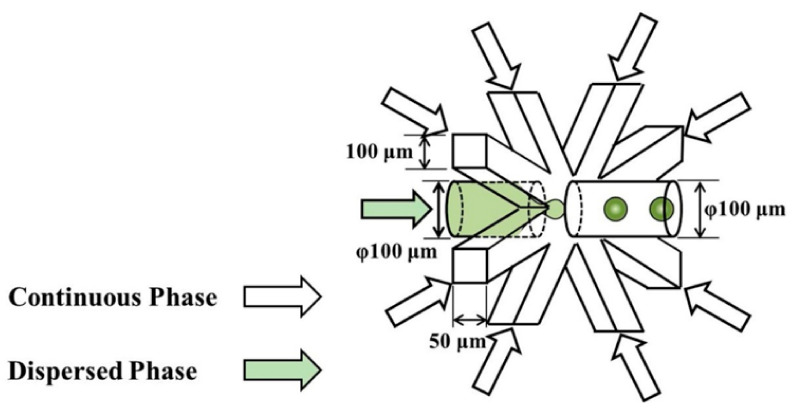
Schematics and dimensions of the microfluidic device designed by Nozaki et al. Reprinted from an open-access source [86].

Another design of a 3D microfluidic device is presented by Nozaki et al. [86], who have developed a platform in which the dispersed phase is broken up by the shear stress of the continuous phase coming from eight directions (Figure 26). The device has one round inlet for the dispersed phase, eight rectangular inlets for the continuous phase, and one round outlet for observation and collection of the generated droplets. Compared to conventional devices, this new design allowed the fabrication of smaller droplets, indicating its excellent performance.

**Figure 27:**
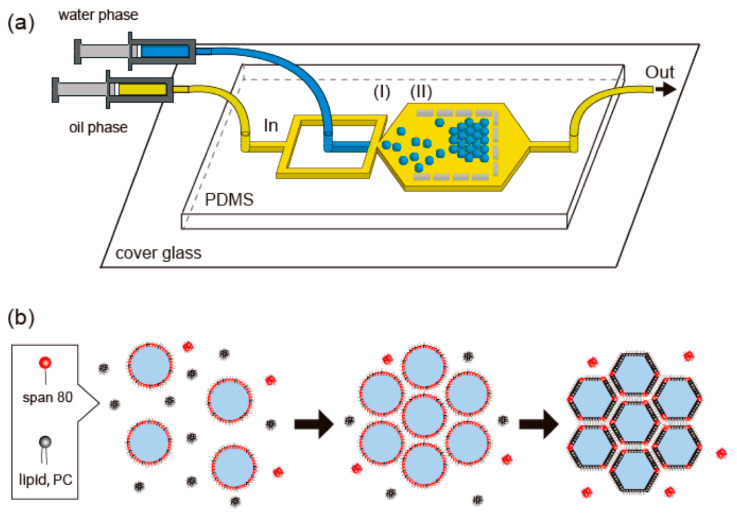
Schematic representation of (**a**) a microfluidic device for preparing the close-packed honeycomb pattern of assembled droplets in a two-dimensional space [(**I**)—droplets are generated via flow-focusing; (**II**)—droplets are trapped and assembled in a U-shaped chamber] and of (**b**) droplet interface bilayer (DIB) formation among droplets. Span 80 molecules (red), having a higher adsorption rate than lipid 1,2-dioleoyl-sn-glycero-3-phosphocholine (PC, black), initially accumulate on the droplet surface. Replacement of Span 80 with PC initiates DIB formation and surface adhesion among droplets. Reprinted from an open-access source [87].

A uniquely-designed microfluidic system is proposed by Fujiwara et al. [87]. The authors approached microfluidic synthesis methods for generating monodispersed droplets and assembling them into droplet interphase bilayers (DIB) with a honeycomb pattern (Figure 27). This was achieved by injecting two surfactants with different adsorption rates on the droplet surface, namely sorbitan monooleate (Span 80) and 1,2-dioleoyl-sn-glycero-3-phosphocholine (PC). The chip configuration and synthesis method allowed for the formation of a honeycomb pattern of DIB-bounded droplets in a single step.

**Figure 28:**
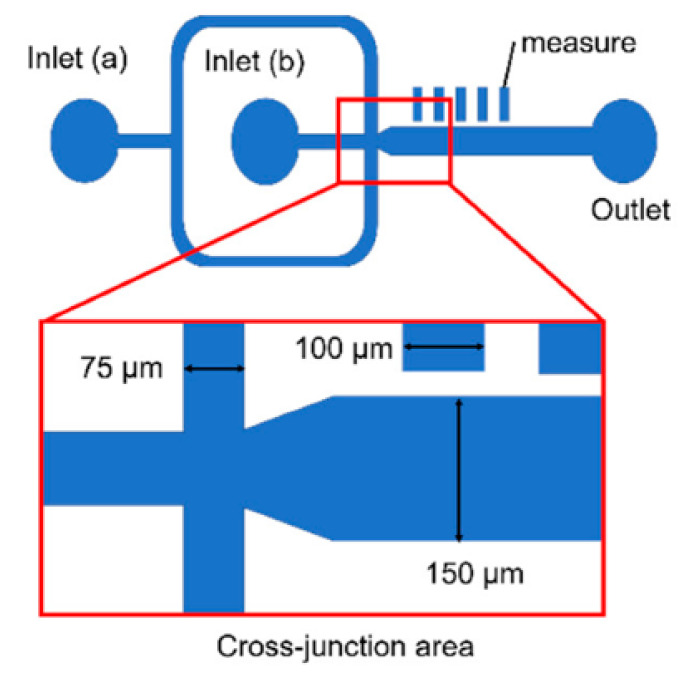
Schematic diagram of channel design for the droplet generation device used by Hattori et al. The widths of the main channel and inlet channels were 150 and 75 μm, respectively, and the channel depth was 100 μm. Reprinted from an open-access source [88].

Another interesting configuration for droplet formation is offered by Hattori et al. [88], who have used the device illustrated in Figure 28 for generating droplets of five common organic solvents (i.e., toluene, chloroform, methanol, tetrahydrofuran, and dimethyl sulfoxide). The dispersed phase solution was delivered through inlet (a), while the continuous phase was injected through inlet (b). The channel possessed a tapered shape at the cross-junction area to facilitate droplet formation. Moreover, several rectangles have been included adjacent to the main channel to allow the measurement of droplet size.

### 4.9. Three-Dimensional Printed Configurations

Three-dimensional printing technology has spectacularly evolved over the years, becoming suitable for more and more applications [89]. Given the fact that it allows for building complex structures with customized geometries in a simple, fast, and low-cost manner, 3D printing has increasingly been regarded as an advantageous method for producing microfluidic platforms. Compared to traditional manufacturing techniques, 3D printing offers a flexible fabricating alternative with fewer steps, suitable for rapid prototyping, while also enabling on-demand production of parts from different materials with high microscale precision [2,90,91,92,93,94]. Moreover, the flexibility of direct 3D printing also facilitates linking micromixer modules, increasing the versatility and functionality of final devices [37,95,96]. Nonetheless, when designing synthesis platforms, special caution must be taken for choosing solvent-resistant materials, which are hard to 3D print. Another potential drawback of 3D printing resides in creating channel surface roughness depending on the resolution of utilized equipment, which may further impact the intended chemical synthesis. However, the surface roughness of the printed material can be regulated by adjusting the size of nozzles or droplets [97,98].

Given the versatility of 3D printing, it is no surprise that numerous studies have tackled this fabrication possibility, leading to the construction of customized microfluidic chips with distinct configurations. For instance, Vasilescu et al. [37] have reported the production of 3D complex microchannel designs for continuous and efficient mixing and production of antibody micro/nanoparticle conjugates. Opting for his method, the authors managed to reduce associated costs and time, simplify the fabrication process, and limit post-processing.

The advantage of minimal to no post-processing was also taken into account by Castiaux et al. [99], who have printed channels ranging in cross-sections from 0.6 cm × 1.5 cm to 125 μm × 54 μm via commercially available PolyJet printers. This fabrication method allows for complex geometries to be designed, printed, and in use in less than 2 h.

Beauchamp et al. [93] have also created microfluidic features via 3D printing. The researchers achieved the fabrication of structures with sub-100 μm external and internal positive and negative resolution features, concluding that even smaller trapping devices can be obtained with more customization of printer control, resin development, and higher resolution projectors.

In another study conducted by Razavi Bazaz et al. [100] was realized the bonding fabrication and bonding of 3D printed devices with high-quality finishing to a transparent polymethyl methacrylate (PMMA) sheet. The desired structure and geometry of the microchannels were created via a high-resolution DLP/SLA 3D printer. Specifically, the scientists managed to manufacture a spiral microchannel with a right-angled triangular cross-section, a configuration that is theoretically impossible to obtain by photolithography.

Alternatively, Ruiz et al. [101] have reported the fabrication of hybrid microfluidic devices incorporating hard and soft materials by the use of an SLA printer for the rigid component (i.e., PMMA) and an FDM printer for the flexible component (i.e., polyurethane). In this manner, the authors designed a finger-actuated pump, a microfluidic quick connect component, and a microfluidic reactor chip with screw-seal sample inlet ports.

**Figure 29:**
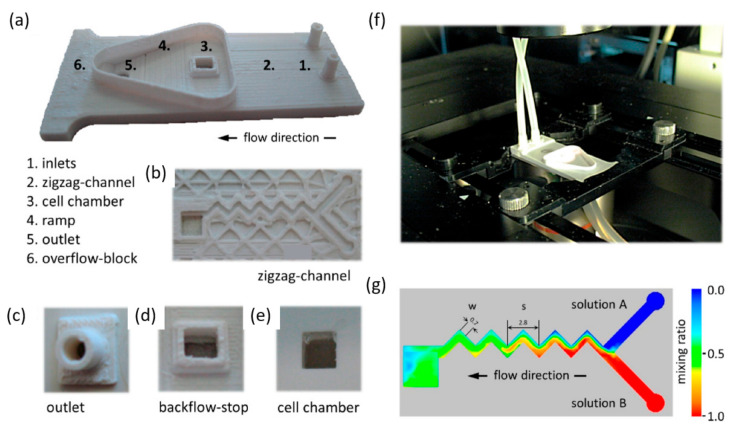
Three-dimensional printed microfluidic platform and simulation of fluid dynamics. (**a**) 3D printed lab-on-a-chip platform with inlets (1), zigzag-shaped microfluidics channel (2), cell chamber (3), ramp (4), overflow block (5), and outlet (6). (**b**) View onto the microfluidic zigzag-shaped channel of an incompletely printed chip. (**c**) Outlet for connection with drainage tubing. (**d**) Top view of the cell chamber, surrounded by a backflow stop to support an outward-directed, unidirectional flow of perfused solution. (**e**) Bottom view of the cell culture chamber through a glass window, mounted with a biocompatible silicone elastomer. (**f**) 3D printed lab-on-a-chip platform with connected tubing for functional imaging using a high-content microscope. (**g**) Dimensions of the zigzag-shaped microchannel for generation of homogeneous mixtures, integrating a “Y” junction, with w, the width of the zigzag channel and s, the linear length of the periodic step. False-color representation of simulated fluid dynamics within the platform for two different solutions, “solution A” (blue) and “solution B” (red). The green color inside the cell chamber indicates a mixing ration of approximately 0.5, and thus demonstrates homogeneous distribution of efficiently mixed solutions. Reprinted from an open-access source [102].

An interesting device was also realized by Aschenbrenner et al. [102], who employed 3D printing of acrylonitrile butadiene styrene to create ultra-low-cost microfluidic platforms. The design is made of two 3D printed parts, namely the main compartment and an outlet connector for drainage tubing (Figure 29). The main advantages of this chip are its light weight (<5 g), small dimensions (20 mm × 49 mm), and cheap fabrication (<1 €).

**Figure 30:**
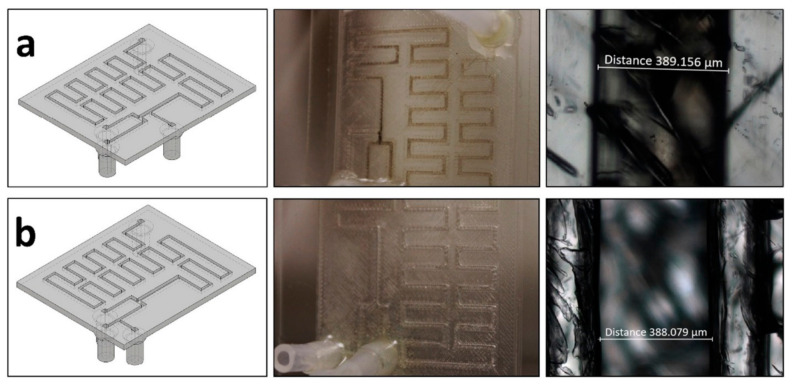
(**a**) (**Left**) Bottom view of the device used to synthesize nanoparticles with the mineral oil inlet put after the reactants. (**Center**) Top view photograph of the actual microfluidic chip showing fouling along the channels. (**Right**) Optical microscopy of the bottom of the channel showing a darker color due to fouling. (**b**) (**Left**) Same as (**a**) but this device presents the mineral oil inlet between the reactants flows. (**Center**) Same as (**a**) but this device presents no fouling along the channels. (**Right**) Same as (**a**) but this time there is no darker coloration inside the channel due to fouling. Reprinted from an open-access source [103].

A different design is proposed by Bressan et al. [103], who have accomplished the generation of a microfluidic platform with low cost and easy fabrication. The authors made a chip from poly(lactic acid) and PMMA, creating transparent microfluidic channels for the continuous-flow synthesis of silver and gold nanoparticles. The device comprised three inlets, one for mineral oil and two for the reactants, and one outlet from where nanoparticles could be collected (Figure 30).

3D printing can also be used not only for developing whole microfluidic platforms but also for fabricating auxiliary components for such systems. For instance, Xu et al. [104] have employed a UV-assisted coaxial printing method for manufacturing microfluidic connectors. In more detail, the researchers fabricated hollow microscale connectors using water as a sacrificial layer and a UV-curable adhesive for shell formation under UV irradiation. On a different note, Van den Driesche et al. [105] utilized 3D printing techniques to create microfluidic chip holders. The authors realized the fluidic sealing between the chip and holder by placing O-rings, partly integrated into the 3D-printed structure, while the electric connection of bonding pads located on microfluidic chips was accomplished by using spring-probes fitted within the printed holder.

### 4.10. Geometries Overview

Overall, there is an increasing interest in designing microfluidic chips and optimizing their geometry to create products with fine-tuned properties. In this respect, numerous studies have focused on fabricating microfluidic devices, performing numerical simulations, analyzing fluid movement, and testing the developed microchips in experimental syntheses. Researchers worldwide managed to exploit the advantages of microfluidic technology for generating microdroplets [44,53,59,72,80,85,86,87,88], porous films [45], double emulsion droplets [55], nanofibers [69], microcapsules [82,83], and micro- [32,56,57,58,70,77,82] and nanoparticles of various compositions, sizes, shapes and morphologies [6,42,50,52,54,60,61,65,66,68,74,76,78,84].

Even though not all the presented devices produced materials with dimensions in the nano range, these platforms were worth mentioning as they can be further adjusted (in terms of geometry, channel dimensions, and process parameters) for the synthesis of nanoparticles in future investigations. However, for the studies that have already managed to generate nanoparticles, Table 1 briefly summarizes information concerning device type, synthesized particles, process parameters, and product properties.

## 5. Conclusions and Future Perspectives

To summarize, microfluidic devices hold great promise in nanoparticle synthesis, benefiting from versatility in design and fabrication. The wide range of available and emerging geometries represents an encouraging factor for choosing microfluidics methods over conventional synthesis techniques for obtaining high-quality nanomaterials in shorter times and with lower manufacturing costs. Moreover, 3D printing is an important asset in the on-demand production of microfluidic devices with customized configurations, high-resolution features, and even hybrid structures.

**Figure 31:**
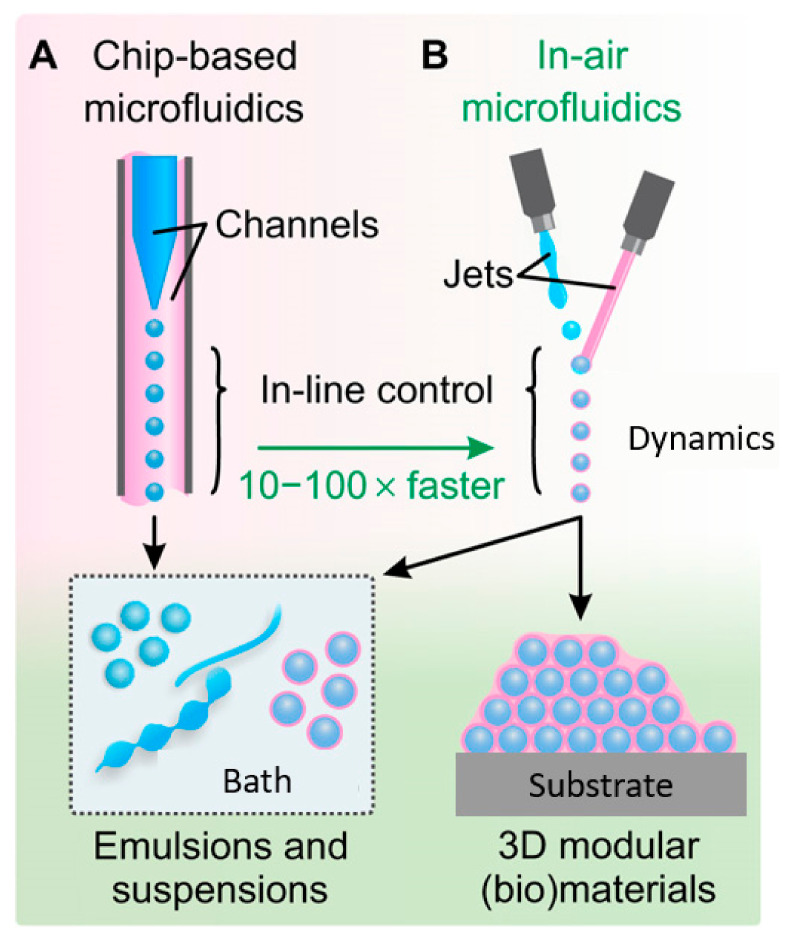
IAMF concept. (**A**) A chip design where droplets (blue) are transported by a coflow (pink). (**B**) IAMF relying on jet ejection and coalescence into air. When combining reactive, solidifying microjets, IAMF also enables on-the-fly production and direct deposition of microparticles into 3D multiscale modular (bio)materials. Reprinted from an open-access source [107].

In addition to the described on-platform synthesis possibilities, interesting prospects may arise from “in-air microfluidics” (IAMF). This method assumes the formation of droplets, fibers, and particles without the need for a chip. The desired materials are obtained in-flight and deposited into 3D constructs with modular internal architecture (Figure 31). Instead of using microchannels, IAMF combines micrometer-sized liquid jets in midair, retaining the processing capacity of chip-based synthesis while enabling orders of magnitude faster production and thus higher throughput [106,107].

Furthermore, investigations of chemical syntheses in various microreactors configurations may also represent an important base for designing chips for other purposes. Specifically, fluid dynamics analyses may be of good use for researchers aiming to fabricate microfluidic platforms for emerging biomedical applications, including non-invasive diagnosis devices [108,109,110,111,112], cell culture media (organs-on-a-chip) [113,114,115,116,117], and precise drug-delivery systems [118,119,120,121].

In conclusion, microfluidics technology has gained a lot of attention from scientists worldwide, bringing new avenues in synthesizing nanomaterials. As it is still in its infancy, microfluidics can benefit from spectacular evolution in terms of chip configurations through interdisciplinary research and advancements in interconnected science fields.

## Figures and Tables

**Table 1:** Overview of discussed microfluidic devices for nanoparticles synthesis.

Device	Synthesized Nanoparticles	Process Parameters	Product Properties	Reference
Y-type microreactor	Curcumin-loaded liposomes	Flow rate ratio—1:1 (organic: aqueous) Total flow rates—1 and 3 mL/min	Mean particle size—~200 nm Encapsulation efficiency—99.9%	[6]
Flow-focusing microreactor	PEG—crosslinked HA nanoparticles	Temperature—4 °C Flow rate ratio—0.27 Thiol | vinyl sulfone groups ratio—0.0011	Mean particle size—150 ± 25 nm	[52]
Flow-focusing microreactor	Silver nanoparticles	Aqueous phase flow rate—14 μL/min Oil phase flow rate—80 μL/min	Particle size range—6.2–34.2 nm (increasing with heating time)	[54]
Flow-focusing microreactor	Magnetite nanoparticles	Side inlet channels flow rate—150 mL/h Middle channel flowrate—varied from 20 to 60 mL/h	Particle size <10 nm Zeta potential—ranged from −72.54 to −4.87 mV	[50]
S-shaped micromixer	Gold nanobipyramids	Total estimated flow rate—ranged from 360 to 520 μL/min	Average length range—134–145 nm Average width range—44–48 nm (increasing with the increase in silver nitrate flow rate)	[61]
S-shape micromixer	PtFeCu/C nanocatalysts	Solvent flow rate-60 mL/h	Average particle diameter depending on solvent: PEG200—1.8 ± 0.3 nm PEG400—2.2 ± 0.3 nm PEG 600—1.7 ± 0.3 nm Water—4.1 ± 0.7 nm EG—3.1 ± 0.4 nm	[4]
Staggered herringbone micromixer	Chitosan (CS)/sodium tripolyphosphate (TPP)	CS/TPP mass ratio—ranged from 5:1 to 8.83:1 Total flow rate—ranged from 5 to 12 mL/min	Average hydrodynamic diameter—ranged from 40 to 400 nm Average Zeta potential—ranged from +18.9 ± 0.6 to +34.6 ± 1.0	[65]
Staggered herringbone micromixer	Metformin and garcinol-loaded niosomes	Total flow rate—ranged from 5 to 12 mL/min	Average particle diameter depending on flow rate ratio (aqueous:organic) and solvent: 1:1—Span-60—<1 μm 1:1—Tween-20—>1 μm 3:1—Span-60—100–150 nm 3:1—Tween-20—100–150 nm 5:1—Span-60—100–150 nm 5:1—Tween-20—<100 nm	[66]
Swirl micromixer	Curcumin-loaded liposomes	Flow rate ratio—3:1 (aqueous:organic) Total flow rate—ranged from 4 to 320 mL/min Reynolds number—ranged from 115.2 to 9217.3 (increased with increasing total flow rate)	Average particle size—ranged from 50.2 to 133.9 nm	[42]
Combined geometry device	Ficin capped gold nano clusters	Temperature—65 °C Oil phase flow rate—33.0 μL/min Aqueous flow rate—ranged from 8.0 to 25.0 μL/min	Average particle size: 5.6 ± 1.0 nm	[74]
Combined geometry device	Liposomes	Lipid solution flow rate: 3 and 4.5 mL/h Aqueous solution flow rate: 30 mL/h	Diameter range: 217–274 nm	[76]
Combined geometry device	PEGylated PLGA nanoparticles loaded with fluorescent dyes	Flow rate ratio—4:6 (organic: aqueous)	Average particle size—~100 nm	[78]
Combined geometry device	Gold nanoparticles	Helium flow rate—1.0 SCCM Gold precursor solution flow rate—0.05 mL/min	Unform particle size Single crystal structure	[84]
3D printed device	Silver nanoparticles	Temperature—20 °C Reactants flow rates—30 and 120 μL/min	Average particle size—ranged from 5 ± 2 nm to 8 ± 3 nm	[103]
3D printed device	Gold nanoparticles	Temperature—90 °C Reactants flow rates—40 and 100 μL/min	Average particle size—ranged from 20 ± 9 to 34 ± 12 nm	[103]

## Data Availability

Not applicable.
